# Effects of temperature alteration on viscosity, polymerization, and in-vivo arterial distribution of N-butyl cyanoacrylate-iodized oil mixtures

**DOI:** 10.1007/s11604-021-01143-3

**Published:** 2021-06-09

**Authors:** Takahiko Mine, Daisuke Yasui, Hidemasa Saito, Tatsuo Ueda, Taro Yokoyama, Shinpei Ikeda, Shohei Mizushima, Seigoh Happoh, Shin-ichiro Kumita

**Affiliations:** 1 Department of Radiology, Nippon Medical School Chiba Hokusoh Hospital, 1715 Kamagari, Inzai, Chiba 270-1694 Japan; 2 Department of Radiology, Nippon Medical School, 1-1-5 Sendagi, Bunkyo-ku, Tokyo, 113-8603 Japan

**Keywords:** NBCA, *n*-butyl cyanoacrylate, Polymerization, Temperature, Viscosity

## Abstract

**Purpose:**

Temperature alteration can modify the polymerization of *n*-butyl cyanoacrylate (NBCA)-iodized oil mixtures during vascular embolization; its effects on viscosity, polymerization time, and intra-arterial distribution of the NBCA-iodized oil mixture were investigated.

**Materials and methods:**

In vitro*,* the viscosities of NBCA, iodized oil, and NBCA-iodized oil mixtures (ratio, 1:1–8) were measured at 4–60 ºC using a rotational rheometer. The polymerization times (from contact with blood plasma to stasis) were recorded at 0–60 ºC using a high-speed video camera. In vivo, the 1:2 mixture was injected into rabbit renal arteries at 0, 20, and 60 ºC; intra-arterial distribution of the mixture was pathologically evaluated.

**Results:**

The mixtures’ viscosities decreased as temperature increased; those at 60 ºC were almost four to five times lower than those at 4 ºC. The polymerization time of NBCA and the 1:1–4 mixtures increased as temperature decreased in the 0–30 ºC range; the degree of time prolongation increased as the percentage of iodized oil decreased. The 0 ºC group demonstrated distributions of the mixture within more peripheral arterial branches than the 20 and 60 ºC groups.

**Conclusion:**

Warming reduces the mixture’s viscosity; cooling prolongs polymerization. Both can be potential factors to improve the handling of NBCA-iodized oil mixtures for lesions requiring peripheral delivery.

**Secondary abstract:**

Temperature alteration influences the polymerization time, viscosity, and intra-arterial distribution of NBCA-iodized oil mixtures. Warming reduces the viscosity of the mixture, while cooling prolongs polymerization.

## Introduction

*N*-butyl cyanoacrylate (NBCA) provides secure vessel occlusion due to its strong adhesive property [1–3]. The mixture of NBCA and iodized oil, which imparts radiopacity and viscosity, has been used in endovascular embolization for various vascular disorders, including cerebral or peripheral arteriovenous malformations [1, 2, 4], traumatic and other sources of idiopathic bleeding [3, 5–7], and aortic intervention-related embolization [3, 8]. Meanwhile, inadequate distribution of the mixture can cause unexpected organ damage [1–3, 5–7, 9, 10]. The extent of the mixture within the vasculature depends upon various factors, including blood flow velocity, vessel anatomy, mixture viscosity, polymerization time, and injection method [1–3, 7, 9–11]. The relation between polymerization time and mixture ratio has been detailed so far [1, 11], and the modification of instruments or preparations to gain better handling of the mixture has also been reported. The blood flow control method using a microballoon catheter, an instrumental technique, is known to efficiently control the extent of the mixture [3, 7, 9]. Further, mixing NBCA with glacial acetic acid or ethanol, a preparation technique, modified the adhesive property of the material and achieved a slow and high amount of infusion [2, 8, 12]. Studies regarding temperature alteration addressed its effect on the mixture viscosity and the polymerization time [3, 13, 14], though the previously evaluated temperature range was limited and a more detailed study may help in better handling of the NBCA-iodized oil mixtures for lesions requiring peripheral delivery.

This study aimed to investigate the effect of temperature alteration on the viscosity, polymerization time, and intra-arterial distribution of the NBCA-iodized oil mixtures with the widest temperature range possible, through in vitro and in vivo experimentations.

## Materials and methods

### In vitro experimentation

#### Viscosity alteration of the NBCA-iodized oil mixtures

Six different ratios of the mixture (NBCA [Histoacryl; B. Braun, Melsungen, Germany]: iodized oil [Lipiodol; Guerbet, Aulnay-sous-Bois, France] = 1:1, 1:2, 1:3, 1:4, 1:6, and 1:8) were prepared from 0.5 mL of NBCA and corresponding amounts of iodized oil, with manual mixing using syringes and a stopcock, and vortexed for 30 s using a vortex mixer (Vortex Genius 3; IKA, Königswinter, Germany). The viscosities of pure NBCA, pure iodized oil, and the mixtures were measured using a rotational rheometer (ARES-G2 Rheometer; TA Instruments, New Castle, DE, USA) at progressively increasing temperatures (4, 10, 20, 30, 40, 50, and 60 ºC); 0.98 mL of the sample was used per rotation, and measurements were performed six times per temperature.

#### Polymerization time of the NBCA-iodized oil mixtures

**Fig. 1:**
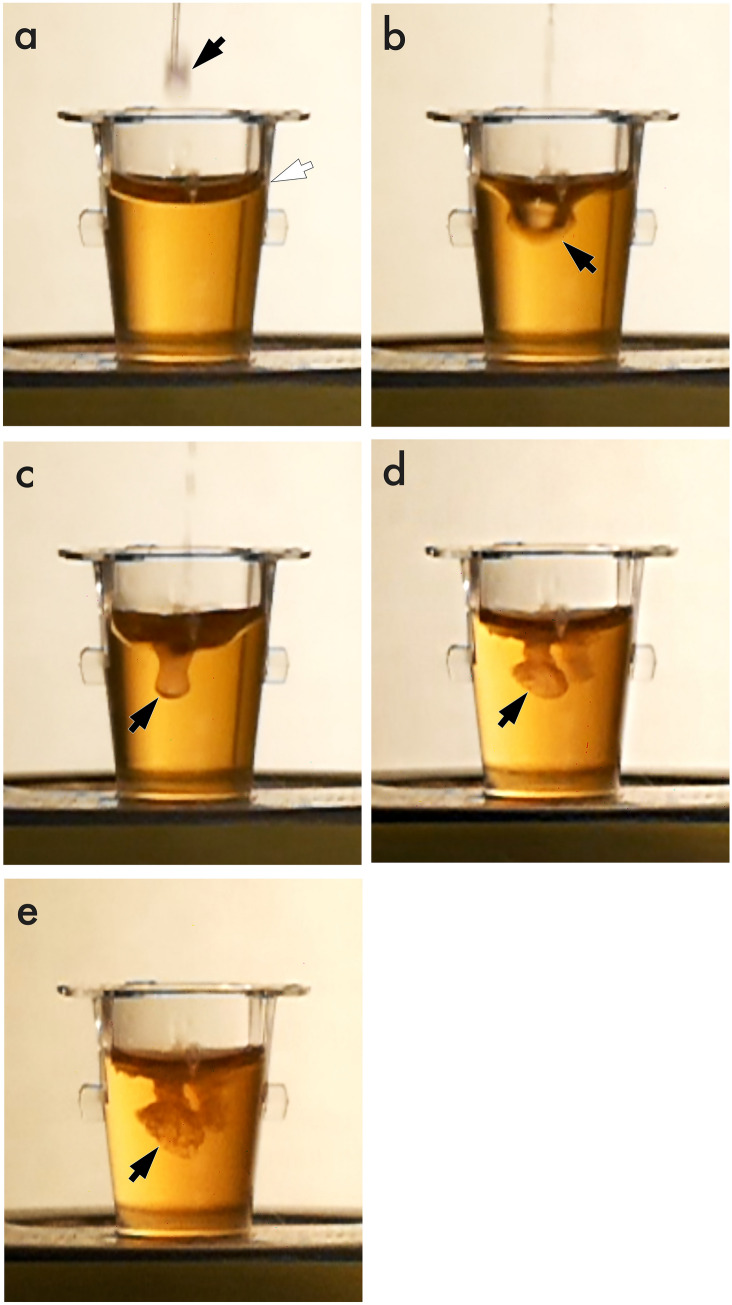
High-speed video images taken during polymerization of the NBCA-iodized oil mixtures. Ten microliters of the mixture, a droplet, (a, black arrow) are dispensed from a micropipette into human blood plasma within a cell culture cluster (a, white arrow). From the mixture’s first contact with blood plasma (b, arrow), the border of the mixture (c-e, arrows) is monitored until stasis.

Six different ratios of the mixture (NBCA: iodized oil = 1:1, 1:2, 1:3, 1:4, 1:6, and 1:8) were prepared from 0.5 mL of NBCA and corresponding amounts of iodized oil with manual mixing. The temperature of pure NBCA and the mixtures was changed from 0 ºC to 60 ºC within an aluminum cup fixed in a water bath (HWA-50A; As One Corp., Osaka, Japan). Ten microliters of the sample were drawn from an aluminum cup using a micropipette (Accumax Smart Pipette; Accumax Lab Devices, Gujarat, India) and dispensed into 1 mL of healthy human blood plasma (centrifuged and 20 ºC) within a cell culture cluster. The infusion was repeated at least four times at each temperature. The polymerization process, from the mixture’s first contact with the blood plasma to stasis (Fig. 1), was recorded using a high-speed video camera (Lumix DC-TZ90; Panasonic, Osaka, Japan) at a rate of 240 frames per second, as reported in a previous study [11].

### In vivo experimentation

#### Study design and embolization procedure

**Fig. 2:**
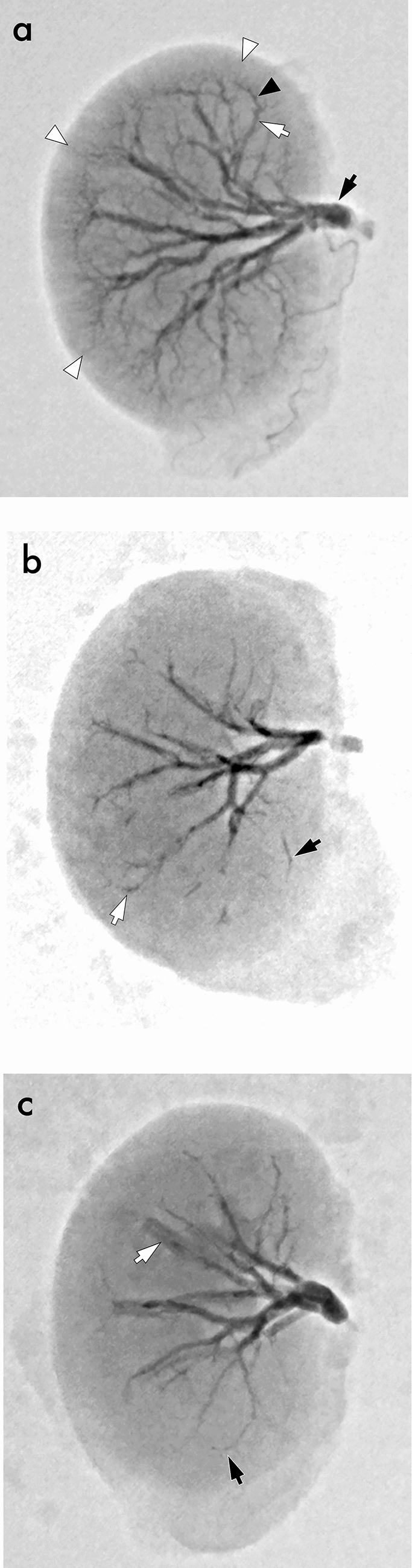
Radiographic images of the NBCA-iodized oil mixture. One whole kidney image of the 0 ºC group (a) contains a continuous mixture within the main trunk of the renal artery (black arrow), interlobar arteries (white arrow), arcuate arteries (black arrowhead), and interlobular arteries (white arrowheads). In the 20 ºC group (b), the mixture in the interlobar arteries (black arrow) and arcuate arteries (white arrow) is fragmental. In the 60 ºC group (c), a continuous mixture is seen up to the arcuate arteries (black arrow), and its volume is higher than that of the 20 ºC group. The contrast medium used in angiography and remaining post-imaging is seen in the renal calyx (c, white arrow).

The in vivo embolization procedure was undertaken after we received approval from the institutional animal care committee. Six adult female Japanese white rabbits weighing 2.56–3.02 kg (mean, 2.86 kg) were used. All procedures were performed under inhalational anesthesia using sevoflurane. A 4-French sheath (Super Sheath; Medikit, Tokyo, Japan) was retrogradely inserted into a surgically exposed common femoral artery under the guidance of X-ray fluoroscopy. The bilateral renal arteries were selected using a cobra-shaped 4-French catheter (Medikit). A 2.2-French microcatheter (SIRABE, 110 cm; Piolax, Yokohama, Japan) was coaxially advanced into the main trunks of the renal arteries. One concentration of the mixture (NBCA: iodized oil = 1:2)—the usual ratio utilized at our clinical practice—was prepared and divided among the three temperature groups: 0 ºC, 20 ºC, and 60 ºC. The 12 renal arteries from the six rabbits were equally divided across the three groups. A mixture for each temperature group was manually injected via a microcatheter, through a 1-cc syringe (Sensitech; TOP Corp., Tokyo, Japan), with as much uniform speed as possible until the renal artery was filled with the mixture. The injection time was recorded using a stopwatch. After embolization, the rabbits were sacrificed using deep anesthesia, and their kidneys were removed. The remaining mixture within the kidneys was confirmed with radiographic images (Fig. 2) using a 76-micron pixel high-resolution detector (Alphenix Hi-Def Detector; Canon Medical Systems, Ota, Japan).

#### Pathological evaluation of the distribution of the NBCA-iodized oil mixtures

**Fig. 3:**
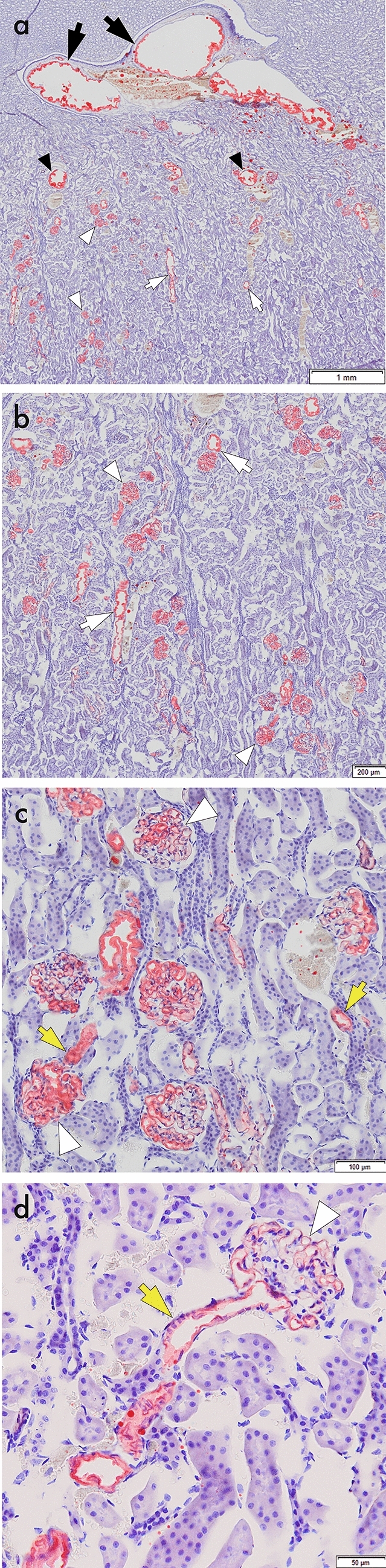
Microscopic images from the pathological evaluation of rabbit kidneys. The deposits containing iodized oil are stained red with Oil Red O. The arterial structures containing the mixture are segmented into interlobar arteries (a, black arrows), arcuate arteries (a, black arrowheads), interlobular arteries (a–b, white arrows), afferent/efferent arterioles (c–d, yellow arrows), and nephrons (a–d, white arrowheads).

Three sections—the renal hilum, medulla, and cortex—were cut from each kidney and stained with Oil Red O. In total, 36 sections, 12 per temperature group, were prepared. Under microscopic examination, the arterial structures were segmented from the proximal to the peripheral area as follows: interlobar arteries, arcuate arteries, interlobular arteries, afferent/efferent arterioles, and nephrons (Fig. 3). For interlobar arteries, arcuate arteries, and nephrons, the ratio of the structures that contained the mixture was calculated relative to the total number of segments. For interlobular arteries and afferent/efferent arterioles, the pure number of arterial structures that contained the mixture was evaluated because these segments were too numerous to determine the total number.

#### Data analysis

An SPSS software package (version 24.0; SPSS Inc., Chicago, IL, USA) was used for the statistical analyses, and mean values were used in each evaluation. The Mann–Whitney U test was used to compare the background of the embolization procedure between the 20 ºC group and the 0 ºC or 60 ºC groups. The Kruskal–Wallis test with Bonferroni correction was used for pathological evaluations. Differences were regarded as significant at *P* < 0.05.

## Results

### In vitro experimentation

#### Viscosity alteration of the NBCA-iodized oil mixtures

**Fig. 4:**
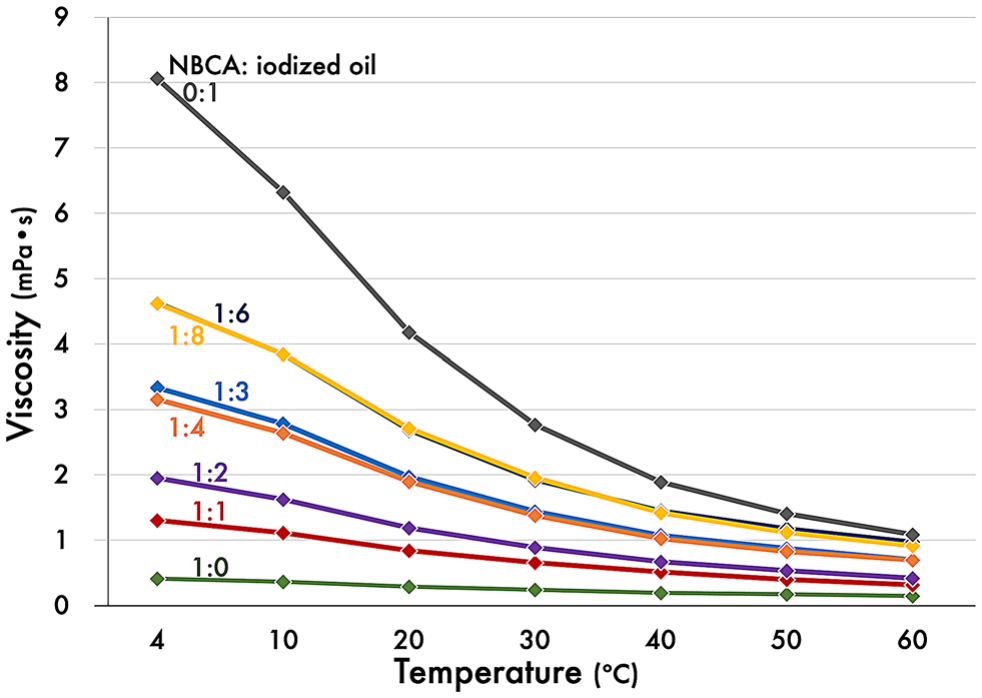
Viscosity alteration of the NBCA-iodized oil mixtures

Viscosity data for NBCA, iodized oil, and the mixtures at various concentrations are shown in Fig. 4. As iodized oil was added, the viscosity of the mixture increased. As temperature increased, the viscosity of all mixtures decreased; those at 60 ºC were almost four to five times lower than those at 4 ºC.

#### Polymerization time of the NBCA-iodized oil mixtures

**Fig. 5:**
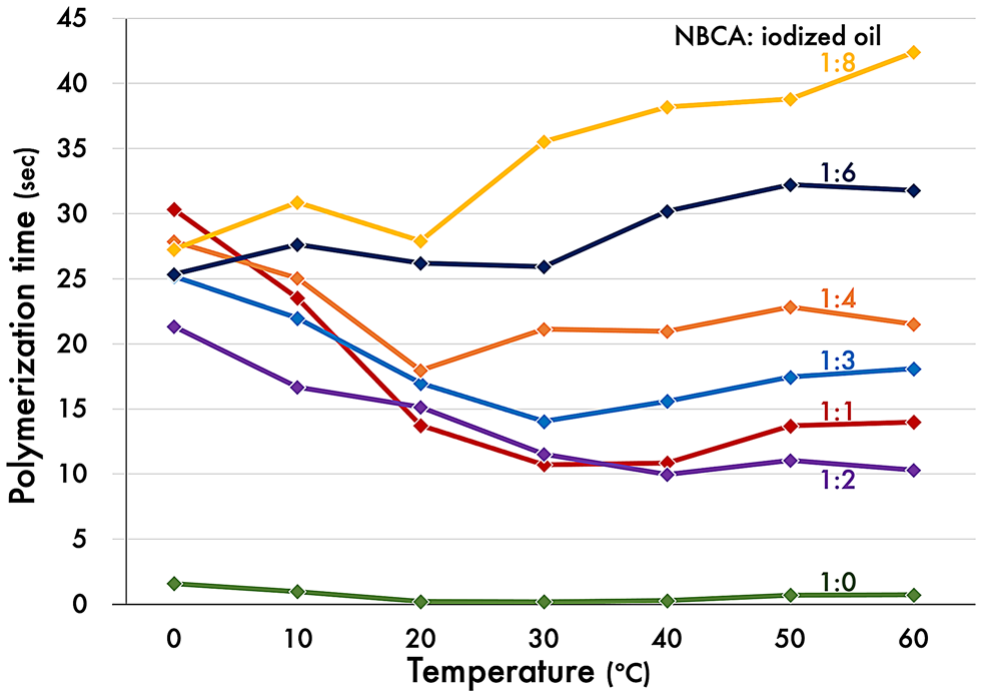
Polymerization time of the NBCA-iodized oil mixtures

Temperature-related changes in polymerization time for each mixture are shown in Fig. 5. For pure NBCA (1:0), the polymerization times at 0 ºC (1.59 s) and 60 ºC (0.72 s) were eight- and three-fold longer, respectively, than that at 20–30 ºC (approximately 0.20 s). For the mixtures with 1:1, 1:2, 1:3, and 1:4 ratios, the polymerization time almost linearly decreased as the temperature decreased from 0 to 30 ºC; meanwhile, a slight increase was observed from 30 to 60 ºC. Notably, the polymerization time particularly increased when we used the 1:1 mixture at 0–30 ºC. For the mixtures with a ratio of 1:6 or 1:8, there were no apparent changes in the polymerization time in the temperature range of 0–30 ºC or 0–20 ºC, respectively, and their polymerization times increased when the temperature increased during 30–60 ºC or 20–60 ºC, respectively.

### In vivo experimentation

#### Background of the embolization procedure

Across the three temperature groups (0 ºC, 20 ºC, and 60 ºC), the kidney volume (7.46 ± 0.42 cm^3^, 8.03 ± 0.27 cm^3^, and 8.03 ± 0.27 cm^3^, respectively), injection times (10.3 ± 2.78 s, 8.00 ± 0.74 s, and 9.84 ± 0.44 s, respectively), volumes of injected mixtures (0.63 ± 0.05 mL, 0.52 ± 0.01 mL, and 0.55 ± 0.03 mL, respectively), and injection speed (0.076 ± 0.01 mL/s, 0.067 ± 0.01 mL/s, 0.057 ± 0.01 mL/s, respectively) did not differ significantly.

#### Pathological evaluation of the distribution of the NBCA-iodized oil mixtures

The pathological evaluation data are shown in Table 1. The ratio of interlobar and arcuate arteries that contained iodized oil to the total number of the same segments in the 0 ºC group was the highest among all three temperatures. It was significantly different from the 20 ºC group (70.8 ± 9.6 vs. 30.2 ± 6.1%, *p* = 0.025). The total number of interlobular arteries and afferent/efferent arterioles that contained iodized oil in the 0 ºC group was significantly higher than that in the 20 ºC and 60 ºC groups (193 ± 59 vs. 1.33 ± 0.5% [*p* = 0.002], 5.35 ± 1.5% [*p* = 0.024], respectively). Distribution of iodized oil within the nephron was only observed in the 0 ºC group (30.3 ± 9.0%).

**Table 1:** Ratio of the total and/or counted number of segmented vasculatures containing the NBCA-iodized oil mixture

Group	Interlobar and arcuate arteries				Interlobular arteries and arterioles			Nephrons		
	Number	ratio (%)	*P-*value		Number	*P-*value		Number	ratio (%)	*P*-value
0 ºC	8.8 ± 1.9	70.8 ± 9.6	^†^0.017	^‡^0.025 (0 vs. 20) ^‡^0.065 (0 vs. 60)	193.2 ± 59.1	^†^0.002	^‡^0.002 (0 vs. 20) ^‡^0.024 (0 vs. 60)	104.8 ± 34.8	30.3 ± 9.0	^†^0.001
20 ºC	3.3 ± 0.9	31.2 ± 6.1			1.3 ± 0.5			0	0	
60 ºC	4.4 ± 1.0	36.6 ± 8.0			4.0 ± 1.5			0	0	

Data are presented as means ± standard errors, ^†^Kruskal–Wallis test, ^‡^Bonferroni correction

## Discussion

Warming decreased the viscosity for all mixtures and pure iodized oil; it may provide a more homogenous and continuous distribution within the vascular bed. This phenomenon could be interpreted based on the Andrade equation about temperature-dependent viscous models for liquids [15]: *η* = *Aexp(Ea/RT)* (*η*, viscosity; *T*, absolute temperature; *A*, pre-exponential factor; *Ea*, activation energy; *R*, gas constant). Bracard et al. were the first to study the effects of temperature on the mixture’s viscosity. Their results showed that viscosity changed within the temperature range of 15–40 ºC [13], and our results suggest that viscosity changes are associated with an even wider range of temperatures. The clinical use of warmed mixtures was recently reported based on their capability of peripheral delivery [3].

Cooling within the temperature range of 0–30 ºC prolonged the polymerization time of pure NBCA and the 1:1–4 mixtures. The polymerization time was most prolonged for pure NBCA and decreased as the proportion of cyanoacrylate within the mixture reduced. Wang et al. demonstrated a relationship between the NBCA polymerization rate and temperature in the range of 20–37.5 ºC, when applied to a polyvinyl alcohol cryogel surface [14]. Although the measured values differ owing to the experimental methods, a similar tendency was observed in our data. Our pathological examination revealed a strong tendency for the mixtures at 0 ºC to distribute within the small branches of the arteries/arterioles. Generally, thermal energy relates directly to motion at the molecular level. Chemical reactions occur more rapidly at high temperatures owing to the increased activation energy [16], according to the equation advocated by Arrhenius: *k* = *Aexp(− Ea/RT)* (*k*, the rate constant; *T*, absolute temperature; *A*, pre-exponential factor; *Ea*, activation energy; *R*, gas constant). During cyanoacrylate’s chemical reaction in the blood, electrical charge instability initiated by contact with anions opens the carbon–carbon double bonds in the monomeric structure, and the monomers transform into polymer chains [2, 3]. Cooling may disturb this process and prolong polymerization, as was observed in the results of in vitro experimentation. The time prolongation observed for pure NBCA at 50–60 ºC might have been owing to deactivation by warming. For both the 1:6 and 1:8 mixtures, the lack of apparent time-related changes during cooling could have been caused by an insufficient volume of cyanoacrylate. The time prolongation observed during warming from 30 to 60 ºC seemed to result from a reduced mixture viscosity.

Our in vivo experimentation demonstrated complex results; the 0 ℃ mixtures with prolonged polymerization delivered mostly peripherally, despite the increased viscosity, and the 60 ℃ mixtures with less viscosity delivered more peripherally than the 20 ℃ mixtures, despite prompted polymerization. The capability of peripheral delivery of the NBCA-iodized oil mixtures depends not only on viscosity and polymerization speed of the mixture but also on various factors, such as diameter and tortuosity of the vessels, blood flow velocity, and injection speed [1–3, 7, 9–11]. We assume that the viscosity of the mixture might especially be more important for distribution in the tortuous arteries but less important in the non-tortuous arteries. Furthermore, we assume that the effect of polymerization speed on mixture distribution may depend on blood flow; it might be more important for distribution in the slow-flow vessels. Rabbit renal arteries are non-tortuous and high-flow vessels; therefore, it remained to be confirmed whether similar results can be obtained in the tortious and/or slow-flow vessels.

Clinically, both decreased viscosity and prolonged polymerization have similar advantages in terms of peripheral delivery of the mixture, although cooling and warming are contrary methods. We presume that these would be helpful for lesions requiring peripheral delivery and/or slow injection to fill the tortuous feeder and the wide volume target simultaneously with the mixture, in a situation where pinpoint-access up to the target is not available, but the target needs a large amount of the NBCA-iodized oil mixtures. For example, a type II endoleak after an endovascular aortic repair frequently requires an approach to the aneurysm via complicated collaterals [8], some types of peripheral arteriovenous malformations have multiple culprit feeders into a huge venous sac [4], and hemoptysis due to chronic lung inflammation is occasionally caused by meandering and wide vasculature [6, 7]. In addition to the mixture ratio, as per their preferences, operators may be able to adjust viscosity and polymerization time by referring to our data. Warming promotes more peripheral delivery of the mixture with a high percentage of iodized oil, and cooling supports slow injection of the mixture with a low percentage of iodized oil. Technically, warming the mixtures may be easier in a preparation, while the adhesive property of the mixture decreases with the increased amount of iodized oil; hence, cooler mixtures with less iodized oil may also be favorable in some conditions with active bleeding and/or coagulopathic conditions.

This preclinical study had several limitations. First, the safety of low-viscosity or cooled mixtures was not evaluated. Extremely peripheral delivery may be associated with surrounding tissue necrosis; hence, strict selection criteria are mandatory in a clinical trial. Second, regarding the in vivo study, our statistical power was limited by the relatively small number of animal subjects. We only used one mixture ratio for the in vivo study to focus on the effect of temperature alteration and not on the concentration to minimize the number of animals sacrificed. Moreover, we could not measure the temperatures of the injected agent at the tip of the microcatheter, the arterial blood of the rabbits, and the renal parenchyma; these parameters could affect the behavior of the mixtures in vivo.

In conclusion, temperature alteration correlated with the NBCA-iodized oil mixtures’ viscosities and polymerization times. Warming and cooling are opposing maneuvers; however, both could be key to improving the handling of the NBCA-iodized oil mixtures for lesions requiring peripheral delivery. Further experimental and clinical studies to corroborate each advantage according to various hemodynamic and anatomical conditions are warranted.

## References

[1] Brothers MF, Kaufmann JC, Fox AJ, Deveikis JP (1989). n-Butyl 2-cyanoacrylate–substitute for IBCA in interventional neuroradiology: histopathologic and polymerization time studies. Am J Neuroradiol.

[2] Gounis MJ, Lieber BB, Wakhloo AK, Siekmann R, Hopkins LN (2002). Effect of glacial acetic acid and ethiodized oil concentration on embolization with N-butyl 2-cyanoacrylate: an in vivo investigation. Am J Neuroradiol.

[3] Mine T, Ueda T, Yasui D, Mizushima S, Kumita SI (2020). Embolization using warmed glue via the triaxial microballoon occlusion system for various vascular disorders. Diagn Interv Radiol.

[4] Mine T, Murata S, Nakazawa K, Onozawa S, Ueda T, Kumita S (2013). Novel endovascular techniques to control in-outflow with dual approach for large pelvic arteriovenous malformation. J Vasc Surg Venous Lymphat Disord.

[5] Mine T, Murata S, Nakazawa K, Onozawa S, Ueda T, Miyauchi,  (2013). Glue embolization for gastroduodenal ulcer bleeding: contribution to hemodynamics and healing process. Acta Radiol.

[6] Shimohira M, Hashimoto T, Abematsu S, Hashizume T, Nakagawa M, Ozawa Y (2015). Triaxial system in bronchial arterial embolization for haemoptysis using N-butyl-2-cyanoacrylate. Br J Radiol.

[7] Mine T, Matsumoto T, Hayashi T, Tomita K, Masuda K, Kawashima M (2018). A stepwise embolization strategy for a bronchial arterial aneurysm: proximal coil and distal glue with the optional use of a microballoon occlusion system. Cardiovasc Intervent Radiol.

[8] Nakai M, Ikoma A, Loffroy R, Midulla M, Kamisako A, Higashino N (2018). Type II endoleak model creation and intraoperative aneurysmal sac embolization with n-butyl cyanoacrylate-lipiodol-ethanol mixture (NLE) in swine. Quant Imaging Med Surg.

[9] Hamaguchi S, Lohman BD, Ogawa Y, Arai Y, Hashimoto K, Matsumoto J (2015). Preliminary findings of arterial embolization with balloon-occluded and flow-dependent histoacryl glue embolization in a swine model. Jpn J Radiol.

[10] Yonemitsu T, Kawai N, Sato M, Sonomura T, Takasaka I, Nakai M (2010). Comparison of hemostatic durability between N-butyl cyanoacrylate and gelatin sponge particles in transcatheter arterial embolization for acute arterial hemorrhage in a coagulopathic condition in a swine model. Cardiovasc Intervent Radiol.

[11] Takasawa C, Seiji K, Matsunaga K, Matsuhashi T, Ohta M, Shida S (2012). Properties of N-butyl cyanoacrylate-iodized oil mixtures for arterial embolization: in vitro and in vivo experiments. J Vasc Interv Radiol.

[12] Kawai N, Sato M, Minamiguchi H, Ikoma A, Sanda H, Nakata K (2012). Basic study of a mixture of N-butyl cyanoacrylate, ethanol, and lipiodol as a new embolic material. J Vasc Interv Radiol.

[13] Bracard S, Macho-Fernández JM, Wang X, Anxionnat R, Picard L (1998). Influence of temperature on embolisation with cyanoacrylate. Interv Neuroradiol.

[14] Wang BH, Boulton M, Lee DH, Pelz DM, Lownie SP (2018). A systematic characterization of the factors influencing polymerization and dynamic behavior of n-butyl cyanoacrylate. J Neurointerv Surg.

[15] Andrade END, C.  (1930). The Viscosity of liquids. Nature.

[16] Andersen JM, Mack J (2017). Decoupling the Arrhenius equation via mechanochemistry. Chem Sci.

